# Clinical validation of REALQUALITY RQ-HPV Screen according to the international guidelines for human papillomavirus DNA test requirements for cervical screening

**DOI:** 10.1186/s12985-018-0965-z

**Published:** 2018-03-20

**Authors:** Michela Iacobellis, Cecilia Violante, Gabriella Notarachille, Angela Simone, Rosa Scarfì, Giuseppe Giuffrè

**Affiliations:** 1 Cytopathology Department of the Hospital Di Venere, Bari, Italy; 2 Laboratory of Molecular Biology Applied to Pathologic Anatomy, Department of Human Pathology in Adult and Developmental Age “G. Barresi”, University of Messina, Messina, Italy

**Keywords:** HPV, DNA testing, Primary cervical cancer screening, International guidelines

## Abstract

**Background:**

According to international guidelines, HPV DNA tests represent a valid alternative to Pap Test for primary cervical cancer screening, provided that they guarantee balanced clinical sensitivity and specificity for cervical intraepithelial neoplasia grade 2 or more severe lesions. The aim of this study was to assess whether REALQUALITY RQ-HPV Screen, a new assay based on real time PCR that targets the E6-E7 region of 14 high-risk human papillomaviruses, meets the criteria for primary cervical cancer screening.

**Methods:**

As required by guidelines, a non-inferiority test was conducted to compare the clinical performance of the test under evaluation with that of a clinically validated reference test (Hybrid Capture 2, HC2). The reproducibility of the device was assessed as well. The clinical samples used to test the hypothesis of non-inferiority and to asses reproducibility comprised 910 and 536 cervical specimens respectively. All specimens were originating from a population-based screening cohort.

**Results:**

The study demonstrates that both the clinical sensitivity and specificity of REALQUALITY RQ-HPV Screen are non-inferior to those of HC2. In addition, an adequate intra- and inter-laboratory reproducibility has been reached by the test.

**Conclusions:**

REALQUALITY RQ-HPV Screen fulfils all the requirements of the international guidelines and can be considered clinically validated for primary cervical cancer screening purposes.

## Background

Cervical cancer screening based on Pap Test represents one of the most successful public health interventions in the last 60 years, having led to the decrease of both cervical cancer incidence and mortality [1]. Recently, randomized control trials, using either of these two high-risk human papillomavirus (hrHPV) DNA testing methods: Hybrid Capture 2 (HC2, QIAGEN, Germany) or GP5+/GP6+ PCR-EIA, have led to recognize them as clinically validated alternatives for use in primary cervical cancer screening, providing 60–70% greater protection compared with cytology [2, 3]. In general, when used for primary screening, any hrHPV DNA test has to guarantee a balanced clinical sensitivity and specificity in order to allow effective detection of cervical intraepithelial neoplasia grade 2 (CIN2) or more severe lesions (>CIN2) and minimize follow up procedures on HPV test-positive women without clinically meaningful disease (<CIN2) [4, 5]. Furthermore, high intra- and inter-laboratory reproducibility is required to ensure reliable performance of the test in clinical practice. In order to guarantee these specification, recent guidelines [6] have proposed a clinical validation strategy based on the comparison of the performance of the assay under evaluation with that of an already clinically validated reference hrHPV test, on samples originating from a population-based screening cohort. In accordance with the above mentioned guidelines, a retrospective study was set up aiming to assess the clinical specificity and sensitivity of REALQUALITY RQ-HPV Screen (AB ANALITICA, Italy) in association with the automated platform GENEQUALITY X120 (AB ANALITICA, Italy). This test, based on real time PCR, targets the E6-E7 region of 14 hrHPV genotypes. The standard comparator test chosen to allow expression of performance in relative terms was HC2, which, besides providing adequate clinical sensitivity and specificity for screening purposes [5–7], is also FDA approved. The reproducibility of the test under evaluation was assessed as well.

## Methods

### Study populations

In order to perform the non-inferiority test, a total of 910 women, who attended for the Cervical Cancer Screening Program in the district of Bari in the period from September 2015 to October 2016, were selected retrospectively from the files in the Cytopathology Department of the Hospital Di Venere, that represents a reference laboratory for cervical cytology. Of the selected women, 73 had histologically confirmed ≥CIN2, 600 had a negative cytology result in this as well as the previous screening round, 192 had a negative cytology result in this as well as the two previous screening rounds and 45 had an abnormal cytology result with <CIN2 histology. Cytologic cervical samples belonging to the first group of women (cases, median age 38 years) were tested to assess the clinical sensitivity of REALQUALITY RQ-HPV Screen for ≥CIN2, while cytologic cervical samples belonging to the second, third and fourth group of women (controls, median age 46 years) were tested to assess its clinical specificity. Samples were selected consecutively within each group of women.

The reproducibility of REALQUALITY RQ-HPV Screen was assessed by testing 536 samples, 168 (31.3%) of which were HC2 positive. These samples, selected to provide an adequate positivity rate according to guidelines [6], were originating from the same screening cohort recruited from September 2015 to October 2016. For intra-laboratory reproducibility, the samples were retested in the Cytopathology Department of the Hospital Di Venere, seven weeks after the first analysis. For inter-laboratory reproducibility, an aliquot of the samples was shipped to the Laboratory of Molecular Biology Applied to Pathologic Anatomy of the Department of Human Pathology in Adult and Developmental Age “G. Barresi” in Messina and tested again 11 weeks after the first analysis. The operators that performed second round DNA testing in Bari and Messina were blinded to the original results and sample cytological/histological status.

### Storage of samples

After collection, samples had been stored in ThinPrep PreservCyt Solution (Hologic, USA) at room temperature throughout the whole duration of this study.

### Hybrid capture 2

HC2 was performed right after cytology on all samples with abnormal cytological results, according to the screening algorithm implemented at Hospital Di Venere. Samples with normal cytology were tested with HC2 between May and October 2016, in parallel with the REALQUALITY RQ-HPV Screen analysis. HC2 allows the detection of 13 hrHPV genotypes (HPV 16, 18, 31, 33, 35, 39, 45, 51, 52, 56, 58, 59, and 68) by hybridization with RNA probes and signal amplification. Briefly, 6 mL of each PreservCyt sample were centrifuged with a conversion buffer, the pellet was resuspended in a denaturing reagent and incubated at 65 °C for 45 min. The hybridization step with the cocktail of RNA probes was performed in a microtiter plate at 60 °C for 60 min. The samples were then transferred to another microtiter plate coated with anti-hybrid antibodies and incubated for 60 min. The immobilized hybrids were detected by adding peroxidase-labelled anti-hybrid antibodies and a chemioluminescent substrate. Light emission was measured semiquantitatively as Relative Light Unit (RLU) in a luminometer. Results were expressed in terms of RLU of the sample divided by the mean RLU of three positive controls, each containing 1.0 pg/mL of HPV DNA. Samples were reported as positive when producing concentration values equal to or greater than 1.0 pg/mL. Positive and negative controls, provided by the manufacturer, were included in each run.

### DNA extraction and amplification with REALQUALITY RQ-HPV Screen

DNA extraction and first round DNA testing with REALQUALITY RQ-HPV Screen were performed between May and October 2016. DNA was extracted from 400 μL of PreservCyt samples using the GENEQUALITY X120 Pathogen kit (AB ANALITICA, Italy) with the fully automated platform GENEQUALITY X120. When required, the platform also performs the setup of the PCR plates and handles the loading of the DNA from the elution plates automatically. Real time PCR with REALQUALITY RQ-HPV Screen was performed, according to manufacturer instructions, on an Applied Biosystems 7500 Fast Dx Real-Time PCR System (Applied Biosystems, CA, USA). This in vitro diagnostic device allows the detection of 12 hrHPVs (31, 33, 35, 39, 45, 51, 52, 56, 58, 59, 66, and 68) as a pool and the individual genotyping of HPV 16 and 18 by real time PCR targeting E6 and E7 regions in HPV genome; human beta-globin gene is detected as well to allow monitoring of both extraction and amplification processes. A ready to use mastermix is provided, containing a cocktail of hydrolysis probes marked with four different fluorochromes: FAM for the hrHPV group, JOE for HPV 16, Cy5 for HPV 18 and ROX for the control gene. A positive control, which allows monitoring fluorescence detection from all channels, is also provided by the manufacturer and was included in each run; a negative control (molecular grade water) was included as well. All samples were amplified once and fluorescence signals from each of the four channels were read independently. Sample positivity was assigned when at least one HPV amplification curve intersected the threshold line, set according to manufacturer instruction, and the corresponding Ct value was < 40. Sample negativity was assigned when no HPV amplification curve intersected the threshold (or the Ct value was ≥40) and the control gene Ct was ≤ 34.

### HPV genotyping

All samples with discordant results were additionally analyzed using AMPLIQUALITY HPV-TYPE EXPRESS v.3.0 (AB ANALITICA, Italy). This genotyping assay, based on the amplification of L1 viral region followed by Reverse Line Blot, is capable of recognizing 40 different HPV types separately [8–10].

### Statistical analysis

To compare the clinical sensitivity and specificity for ≥CIN2 of REALQUALITY RQ-HPV Screen to that of HC2, a non-inferiority score test with a power of at least 80% was performed [6, 11]. The relative sensitivity and specificity thresholds used were 90% and 98% respectively, as proposed in the published guidelines [6]; a *P* value < 0.05 was considered statistically significant. The Cohen’s kappa statistic was used to test intra- and inter-laboratory reproducibilities.

## Results

No samples resulted unsuitable for analysis with REALQUALITY RQ-HPV Screen (i.e. negative for HPV, with control gene Ct > 34).

REALQUALITY RQ-HPV Screen as well as HC2 were positive for the same 71/73 ≥ CIN2 samples, showing an absolute sensitivity of 97.3% (confidence interval [CI] 90.5% to 99.7%) (Table 1). Of the two ≥CIN2 samples that resulted HPV negative, one was histologically classified as adenocarcinoma and the other as CIN2. Using AMPLIQUALITY HPV-TYPE EXPRESS, the CIN2 specimen resulted positive for HPV 73, whereas the adenocarcinoma was confirmed as negative.

**Table 1:** Comparison of the REALQUALITY RQ-HPV Screen and HC2 results on 910 cervical samples

		HC2		
Sample type	REALQUALITY RQ-HPV Screen	Positive	Negative	Total
Cases (≥CIN2)	Positive	71	0	71
	Negative	0	2	2
	Total	71	2	73
Controls (<CIN2)	Positive	66	17	83
	Negative	14	740	754
	Total	80	757	837

REALQUALITY RQ-HPV Screen was negative for 754/837 < CIN2 samples, showing an absolute specificity of 90.1% (CI 87.9% to 92.0%), while HC2 was negative for 757/837 samples with an absolute specificity of 90.4% (CI 88.3% to 92.4%) (Table 1).

The non-inferiority score test demonstrated that both the clinical sensitivity and specificity for ≥CIN2 of REALQUALITY RQ-HPV Screen were non-inferior to those of HC2 (*t* = 2.81, *P* = 0.002 for sensitivity; *t* = 2.04, *P* = 0.021 for specificity).

The intra-laboratory reproducibility regarding the two rounds of test performed in Bari was 99.4% (533/536; CI 98.4% to 99.9%) with a kappa value of 0.987 (Table 2).

**Table 2:** Intra-laboratory reproducibility over time of the REALQUALITY RQ-HPV Screen

	First result		
Second result	Positive	Negative	Total
Positive	167	2	169
Negative	1	366	367
Total	168	368	536

The inter-laboratory reproducibility for the specimens tested in the laboratories of Bari and Messina was 99.6% (534/536; CI 98.7% to 100.0%) with a kappa value of 0.991 (Table 3).

**Table 3:** Inter-laboratory agreement of the REALQUALITY RQ-HPV Screen

	Result at Bari lab		
Result at Messina lab	Positive	Negative	Total
Positive	168	2	170
Negative	0	366	366
Total	168	368	536

When separately considering the three possible outcome for positivity (i.e. HPV 16, HPV 18 and one or more of the 12 hrHPVs detected as a pool), kappa values associated to the intra- and inter-laboratory agreement ranged from 0.931 to 1.000 (Table 4).

**Table 4:** Intra- and inter-laboratory agreement for possible positivity outcome with REALQUALITY RQ-HPV Screen

	Intra-laboratory agreement				Inter-laboratory agreement			
HPV type	Both runs	Run 1 only	Run 2 only	Kappa	Both labs	Lab 1 only	Lab 2 only	Kappa
HPV 16	65	1	2	0.974	66	0	2	0.983
HPV 18	14	0	0	1.000	14	0	2	0.931
hrHPV	116	3	3	0.968	118	1	2	0.984

## Discussion

REALQUALITY RQ-HPV Screen, a novel real time PCR assay targeting E6 and E7 regions in HPV genome, has been assessed in the present study according to Meijer et al. international guidelines [6]. Sample selection, however, was done according to the recent validation strategy proposed in the VALGENT study framework [12] and was based on cytology screening alone instead of HC2 results, either or not combined with cytology, as suggested by Meijer et al. [6]. In particular, to guarantee that samples used for specificity assessment were truly disease free samples, only those belonging to women who had at least two negative subsequent cytology results were selected. Then, again along the lines of the VALGENT protocol, this population was enriched with a subset of women that had an abnormal cytological results, but were confirmed as <CIN2 after referral for colposcopy and biopsy histological examination. This strategy is considered to be more stringent for the selection of specificity samples. On the other hand, being selected only on the base of cytological results, the sensitivity population misses those ≥CIN2 cases which, in a co-testing context (e.g. cytology and HC2 based screening), would derive from a negative cytology, but positive HPV DNA result, potentially favouring a more specific but less sensitive test. Ultimately, both the above mentioned selection strategy might lead to the introduction of small biases, favouring either more sensitive or more specific assays, thus, addressing these last minor pitfalls might be a goal of future validation guidelines [13].

Our findings show that REALQUALITY RQ-HPV Screen performs at least equally well as HC2, as the null hypothesis of inferiority was rejected for both sensitivity (*P* = 0.002) and specificity (*P* = 0.021). Only two ≥CIN2 samples resulted negative for both assays. The genotyping of these two cases confirmed the absence of HPV DNA in the case histologically classified as adenocarcinoma. This finding is in agreement with recent works reporting cases of HPV absence in this type of cancer [14–17]. The other sample resulted positive for HPV 73, a genotype not detectable with both HC2 and REALQUALITY RQ-HPV Screen and reported as a probable high risk type in the IARC classification.

The intra- and inter-laboratory reproducibility of REALQUALITY RQ-HPV Screen was evaluated on 536 samples, 31.3% of which had been tested positive in a reference laboratory using HC2. Cohen’s kappa values associated to intra- and inter-laboratory reproducibility experiments were greater than the minimum required value for a clinical test [6] and proved that the test under evaluation produces reliable results.

REALQUALITY RQ-HPV Screen is a so-called open device compatible with different extraction systems; however, in the context of this study, it was evaluated in association with the fully automated high-throughput platform GENEQUALITY X120, which represents an ideal option for mass screening purposes. Compared to HC2, it also offers the advantages of the co-amplification of the beta-globin gene, to assure good quality of samples, and of the direct genotyping of HPV 16 and HPV 18. This latter capability enables longitudinal genotype specific assessments of persistence in women attending the cervical screening programs, even if this aspect is still a matter of debate [18].

Devices with assay designs more similar to REALQUALITY RQ-HPV Screen than HC2, such as Abbott RealTi*m*e High-Risk HPV (Abbot Molecular, USA) and cobas® 4800 HPV Test (Roche Molecular Diagnostics, USA) have already been validated according to Meijer et al. international guidelines [18, 19] corroborating our observation that a multiplex real time PCR based device can be well suited for the purpose of screening.

## Conclusions

REALQUALITY RQ-HPV Screen fulfils all the requirements of the international guidelines and can be considered clinically validated for primary cervical cancer screening purposes.

## Abbreviations

CIN2: Cervical intraepithelial neoplasia grade 2
HC2: Hybrid Capture 2
hrHPV: high-risk human papillomavirus
